# Multidisciplinary approach to screening and management of children with Fabry disease: practice at a Tertiary Children’s Hospital in China

**DOI:** 10.1186/s13023-021-02136-1

**Published:** 2021-12-14

**Authors:** Qian Shen, Jialu Liu, Jing Chen, Shuizheng Zhou, Yi Wang, Lifei Yu, Li Sun, Liuhui Wang, Bingbing Wu, Fang Liu, Yun Cao, Ying Huang, Jianshe Wang, Chenhao Yang, Daqian Zhu, Yangyang Ma, Zhengmin Xu, Wei Lu, Lili Fu, Wenhao Zhou, Hong Xu

**Affiliations:** 1 Department of Nephrology, Children’s Hospital of Fudan University, National Children’s Medical Center, Shanghai, China; 2 Department of Neurology, Children’s Hospital of Fudan University, National Children’s Medical Center, Shanghai, China; 3 Department of Rheumatology, Children’s Hospital of Fudan University, National Children’s Medical Center, Shanghai, China; 4 Department of Dermatology, Children’s Hospital of Fudan University, National Children’s Medical Center, Shanghai, China; 5 Clinical Genetic Center, Children’s Hospital of Fudan University, National Children’s Medical Center, Shanghai, China; 6 Pediatric Heart Center, Children’s Hospital of Fudan University, National Children’s Medical Center, Shanghai, China; 7 Department of Neonatology, Children’s Hospital of Fudan University, National Children’s Medical Center, Shanghai, China; 8 Department of Gastroenterology, Children’s Hospital of Fudan University, National Children’s Medical Center, Shanghai, China; 9 Center for Pediatric Liver Diseases, Children’s Hospital of Fudan University, National Children’s Medical Center, Shanghai, China; 10 Department of Ophthalmology, Children’s Hospital of Fudan University, National Children’s Medical Center, Shanghai, China; 11 Department of Psychology, Children’s Hospital of Fudan University, National Children’s Medical Center, Shanghai, China; 12 Department of Pathology, Children’s Hospital of Fudan University, National Children’s Medical Center, Shanghai, China; 13 Department of Otolaryngology-Head and Neck Surgery, Children’s Hospital of Fudan University, National Children’s Medical Center, Shanghai, China; 14 Department of Endocrinology and Inherited Metabolic Diseases, Children’s Hospital of Fudan University, National Children’s Medical Center, Shanghai, China; 15 Department of Social Work, Children’s Hospital of Fudan University, National Children’s Medical Center, Shanghai, China

**Keywords:** Fabry disease, Multidisciplinary team, Rare disease, Screening, Dried blood spot, Enzyme replacement therapy, Children

## Abstract

**Background:**

Fabry disease (FD) remains poorly recognized, especially in children in China. Considering the diversity and nonspecific clinical manifestations accompanying with life-threatening aspect of this disease, methods to improve effective screening and management of the suspects are needed. This study aims to explore how it can be done effectively from a multidisciplinary perspective for children with FD at a tertiary children’s hospital in China.

**Methods:**

A multidisciplinary team (MDT) of pediatric FD experts was launched at Children’s Hospital of Fudan University. Children with high-risk characteristics were referred by the MDT screening team using the dried blood spot (DBS) triple-test (α-galactosidase A, globotriaosylsphingosine, *GLA* gene). For newborns who were undergoing genetic testing in the hospital, the *GLA* gene was listed as a routine analysis gene. Evaluation, family screening, and genetic counselling were implemented after screening by the MDT management team.

**Results:**

Before the establishment of the MDT, no case was diagnosed with FD in the hospital. However, twelve months following the MDT program's implementation, thirty-five children with high-risk profiles were referred for screening by DBS triple-test, with a yield of diagnosis of 14.3% (5/35). These 5 diagnosed children were referred due to a high-risk profile of pain accompanied by dermatological angiokeratoma and hypohidrosis (n = 2), pain accompanied by abnormal liver function (n = 1), pain only (n = 1), and unexplained renal tubular dysfunction (n = 1). Two neonates were detected early with *GLA* mutations in the hospital, with a yield of detection of 0.14% (2/1420). Furthermore, another 3 children diagnosed with FD were referred from other hospitals. Family screening of these 10 diagnosed children indicated that 9 boys inherited it from their mothers and 1 girl inherited it from her father. Four of them started to receive enzyme replacement therapy.

**Conclusion:**

Screening and management of children with FD is effective based on a defined screening protocol and a multidisciplinary approach. We should pay more attention to the high-risk profiles of pain, angiokeratoma, decreased sweating, and unexplained chronic kidney disease in children.

## Background

Fabry disease (OMIM 301,500, FD) is an X-linked lysosomal storage disorder (LSD) caused by mutations in the *GLA* gene leading to deficient α-galactosidase A (α-Gal A) activity. This enzyme deficiency results in an accumulation of globotriaosylceramide (GL-3) and globotriaosylsphingosine (deacylated form of GL-3, lyso-GL-3) [1]. Phenotypes vary from the “classic” phenotype, with pediatric onset and multiorgan involvement, to “nonclassical” later onset, a predominantly cardiac phenotype. Young patients may initially experience pain, hypohidrosis and gastrointestinal symptoms. Other manifestations of FD, such as renal and cardiac disease, manifest later in adolescence or adulthood [2]. Newborn screening identified a surprisingly high frequency of males with FD (~ 1 in 1,250) [3].

Currently, numerous *GLA* mutations are reported in gene mutation databases [4]. Enzyme replacement therapy (ERT) and adjunctive treatments can provide significant clinical benefit [5]. Early treatment before the onset of potentially irreversible vital organ pathology is ideal. Asymptomatic children with *GLA* mutations should be followed closely for the development of signs, symptoms, or laboratory changes, which would warrant treatment initiation [6]. A comprehensive care plan should be implemented to guide the management of children with FD.

Although FD has been known for more than a century, this LSD remains poorly recognized, especially in children in China. A typical patient’s odyssey means multiple visits to more than ten different medical specialists before the child achieves a confirmatory diagnosis of FD. On average, this process comes 14–16 years following the onset of the first symptoms [7]. Considering the diversity and “nonspecific” clinical manifestations accompanying with life-threatening aspects of FD, methods to improve effective screening and management of suspect cases are needed.

Testing from dried blood spots (DBSs) is now possible for LSDs, making population screening technically feasible. α-Gal A and lyso-GL-3 analysis and *GLA* sequencing in DBS for the diagnosis of FD were evaluated [8]. α-Gal A activity testing is diagnostic for male patients. However, confirmation of the disease-causing *GLA* mutation is important to help establish the disease phenotype. In female patients, as α-Gal A activity is often found within the normal range, testing consisting of plasma or DBS lyso-GL-3 analysis and *GLA* sequencing provides the greatest sensitivity and specificity [9]. In addition, due to the multiple organ systems affected by FD and the complexity of disease management, it is recommended that multidisciplinary teams (MDTs) be established wherever possible to oversee the management of pediatric patients with FD [5]. The aim of this study was to explore how an effective multidisciplinary perspective and DBS triple-testing (alpha-Gal A, Lyso-GL-3, *GLA* gene) could be used for screening and management of children with FD at a tertiary children’s hospital in China.

## Methods

### MDT establishment

**Fig. 1:**
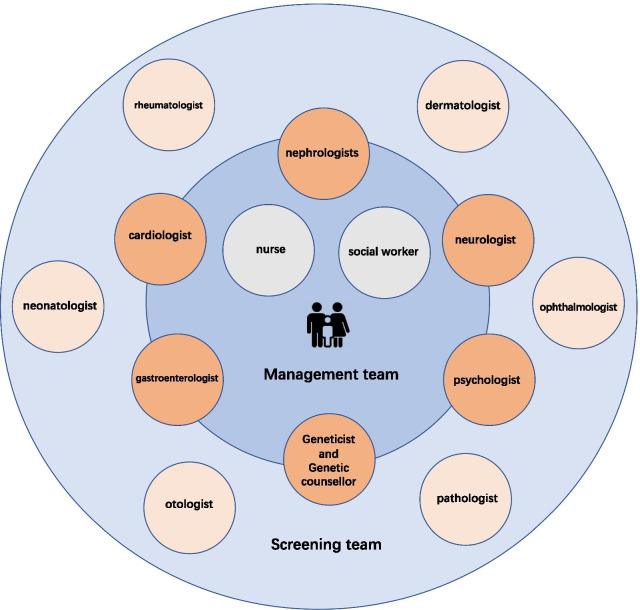
Multidisciplinary team of pediatric Fabry disease

This study was approved by the Ethics Board of Children’s Hospital of Fudan University. In April 2020, an MDT for pediatric FD was launched at Children’s Hospital of Fudan University, including a screening team (nephrologists, neurologist, rheumatologist, cardiologist, gastroenterologist, dermatologist, ophthalmologist, neonatologist, psychologist, otologist, pathologist, geneticist) and management team (nephrologists, neurologist, cardiologist, gastroenterologist, psychologist, genetic counsellor, nurse, and social worker) (Fig. 1). Continuous education was given not only to MDT members but also to other staff in associated departments.

### Approaches for screening, diagnosis, and follow-up

In May 2020, an MDT for pediatric FD established approaches for screening, diagnosis and follow-up. This program started in June 2020. Signed informed consent was obtained from all participants.

Children with high-risk profiles, including accompanying symptoms suggestive of FD (pain presenting as dysesthesia or episodic crisis of burning in hands or feet, dermatological angiokeratoma, anhidrosis or hypohidrosis, corneal verticillata, unexplained chronic kidney disease, unexplained hypertrophic myocardiopathy or heart failure, unexplained early onset stroke, unexplained abdominal pain or intermittent diarrhoea and constipation, and unexplained hearing loss) or family members with FD, were referred by the MDT screening team.

The DBS triple-test (α-Gal A, Lyso-GL-3, *GLA* gene) was used to carry out screening for high-risk children. For boys, a level of α-Gal A determined by multiplexed tandem mass spectrometry (MS/MS) below normal (normal > 2.4 µmol/l/h) was followed by long-range PCR and Sanger sequencing of the *GLA* gene, and the level of lyso-GL-3 was tested by MS/MS. For girls, a level of lyso-GL-3 tested by MS/MS above normal (normal < 1.11 ng/ml) was followed by long-range PCR and Sanger sequencing of the *GLA* gene, and the level of α-Gal A was determined by MS/MS.

For newborns who were at risk of genetic metabolic disorders undergoing genetic testing (whole-exome sequencing or targeted exome sequencing based on exome capture technology) in the hospital [10], the *GLA* gene was listed as a routine analysis gene. A *GLA* pathogenic or likely pathogenic variant interpreted according to ACMG guidelines [11] was followed by measuring the levels of α-Gal A and lyso-GL-3 using DBS.

**Fig. 2:**
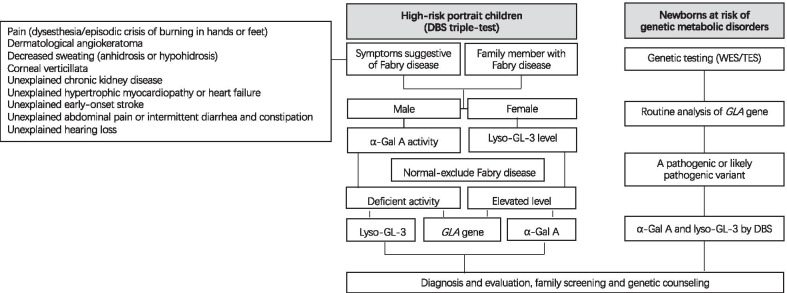
Flowchart for screening, diagnosis, and follow-up of pediatric Fabry disease. DBS, dried blood spots; WES, whole exome sequencing; TES, targeted exome sequencing; α-Gal A, α-galactosidase A; lyso-GL-3, globotriaosylsphingosine (deacylated form of GL-3)

Patients were determined to have a positive test result for FD if they met one of the following criteria [12]: (1) Boys and girls with a *GLA* pathogenic or likely pathogenic variant according to ACMG guidelines, (2) boys with a *GLA* variant of uncertain clinical significance (VUS) and low α-Gal A activity, or (3) girls with a *GLA* VUS and elevated lyso-GL-3 levels. Diagnosis, evaluation, family screening, and genetic counselling were implemented by the MDT management team targeting two aims: (1) how to optimize the evaluation, management, and follow-up of the patient; (2) how to achieve a balance between standardized and individualized treatment. Screening objects and flowchart are shown in Fig. 2.

## Results

### DBS triple-test screening for high-risk children

**Fig. 3:**
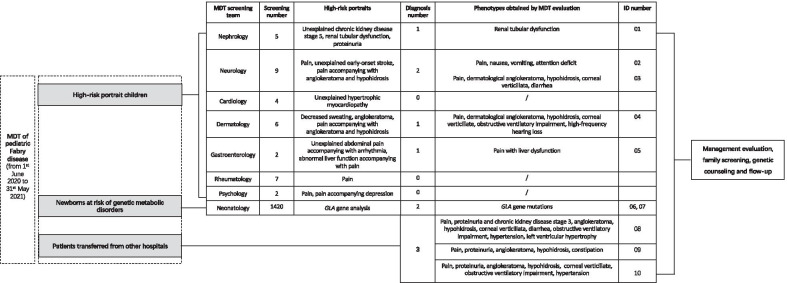
MDT screening and management of children with Fabry disease. MDT, multidisciplinary team; ID, individual identifier

Before the establishment of the MDT, no case was diagnosed with FD in the hospital. However, twelve months following the program's implementation (from 1^st^ June 2020 to 31^st^ May 2021), thirty-five children with defined high-risk profiles were referred for screening with the DBS triple-test. Among these 35 suspected patients, nine were referred by neurologists due to pain only (n = 6), unexplained early-onset stroke (n = 2), or pain accompanied by angiokeratoma and hypohidrosis (n = 1); seven were referred by rheumatologists due to pain (n = 7); six were referred by dermatologists due to decreased sweating (n = 4), angiokeratoma (n = 1), or pain accompanied by angiokeratoma and hypohidrosis (n = 1); five were referred by nephrologists due to unexplained chronic kidney disease stage 5 (n = 2), renal tubular dysfunction (n = 2), or proteinuria (n = 1); four were referred by cardiologists due to unexplained hypertrophic myocardiopathy; two were referred by gastroenterologists due to unexplained abdominal pain accompanied by arrhythmia (n = 1) or abnormal liver function accompanied by pain (n = 1); two were referred by psychologists due to pain accompanied by depression (n = 1) or pain only (n = 1) (Fig. 3).

Five children were diagnosed with Fabry disease by DBS triple-test screening, with a yield of diagnosis of 14.3% (5/35). These 5 children were screened due to high-risk profiles of pain accompanied by dermatological angiokeratoma and hypohidrosis (n = 2), pain accompanied by abnormal liver function (n = 1), pain only (n = 1), and unexplained renal tubular dysfunction (n = 1). MDT evaluation, family screening, and genetic counselling were implemented for these patients (ID 01, 02, 03, 04, 05 in Fig. 3 and Table 1). Four were boys, and family screening indicated that all inherited it from their mothers, and the other was a girl who inherited it from her father. The diagnosis age ranged from 0.7 years old to 16.0 years old, with 0–6.9 years following the onset of the first symptoms.

**Table 1:** Demographic and clinical characteristics of 10 diagnosed FD patients

ID	Age at diagnosis (years)	Age at first symptom (years)	Sex	Proband α-Gal A (normal > 2.4 umol/l/h)	Proband lyso-GL-3 (normal < 1.11 ng/ml)	GLA NM_000169.2 Nucleotide alteration, Protein change	Zygosity (Segregation)	ACMG classification	Phenotypes from pedigree analysis	α-Gal A from pedigree analysis (normal > 2.4 umol/l/h)	Lyso-GL-3 from pedigree analysis (normal < 1.11 ng/ml)	Treatment	
												ERT	Adjunctive treatment
01	0.7	0.7	Male	0.4	32.63	c.195-1G > A	Hemi (F, WT;Aff. M, het)	LP	Pain	1.21	8.24	No	Potassium citrate
02	7.2	3.6	Female	3.7	1.69	c.640-801G > A (also known as IVS4 + 919 G > A)	Het (Aff. F, Hemi; M, WT)	P	Sinus arrhythmia, High left ventricular voltage	1.24	3.43	No	Topiramate
03	11.4	8.4	Male	0.42	100.48	c.277 G > A, p.Asp93Asn	Hemi (F, WT; Aff. M, het)	P	None	0.57	7.60	No	None
04	10.4	3.5	Male	0.41	92.98	c.140G > A, p.W47X	Hemi (F, WT; Aff. M, het)	LP	Pain	NA	6.17	Yes	Oxcarbazepine
05	16.0	16.0	Male	0.23	2.29	c.335 G > A, p.R112H	Hemi (F, WT; Aff. M, het)	LP	None	NA	1.00	No	None
06	0.1	N	Male	1.64	< 0.55	c.257A > T, p.Y86T	Hemi (F, NA; Aff. M, het)	LP	Pain	2.73	2.77	No	None
07	0.3	N	Male	1.42	< 0.55	c.215 T > C, p.M72T (novel variant)	Hemi (F, WT; Aff. M, het)	LP	None	NA	< 0.55	No	None
08	15.6	11.7	Male	0.25	76.06	c.974G > A, p.G325D	Hemi (F, NA; Aff. M, het)	P	None	NA	4.56	Yes	ARB, Carbamazepine, Calcitriol, Ketoacid, Febuxostat, Psychiatry referral
09	13.6	7.5	Male	0.3	104.76	c.100A > C, p.Asn34His	Hemi (F, WT; Aff. M, het)	LP	Pain, Dermatological angiokeratoma, Corneal verticillata, Hypertension, Left ventricular hypertrophy, Diarrhea	NA	ND	Yes	Carbamazepine, Psychiatry referral
10	15.7	4.5	Male	0.26	113.57	c.679C > T, p.Arg227X	Hemi (F, NA; Aff. M, het)	P	Proteinuria	NA	NA	Yes	ACEI, Carbamazepine

α-Gal A, α-galactosidase A; ACEI, angiotensin-converting enzyme inhibitor; ACMG, American College of Medical Genetics and Genomics; Aff., affected; ARB, angiotensin receptor blocker; ERT, enzyme replacement therapy; F, father; FD, Fabry disease; Hemi, hemizygous; Het, heterozygous; ID, individual identifier; Lyso-GL-3, globotriaosylsphingosine (deacylated form of GL-3); LP, likely pathogenic; M, Mother; NA, not available; P, pathogenic; WT, wild type

### Newborn screening by genetic testing

Two neonates (ID 06, 07 in Fig. 3 and Table 1) were found to have *GLA* mutations who were at risk of inherited metabolic disorders and were undergoing genetic testing in the hospital from 1^st^ June 2020 to 31^st^ May 2021, with a yield of detection of 0.14% (2/1420). The levels of α-Gal A were lower than normal, and lyso-GL-3 was in the normal range in these 2 neonates. Both of them were boys, and family screening indicated inheritance from their mothers.

### Refereed patients for MDT management from other hospitals

Three children diagnosed with FD (ID 08, 09, 10 in Fig. 3 and Table 1) were referred from other hospitals for further MDT evaluation and management. All were boys, and family screening indicated that all were inherited from their mothers. The diagnosis ages were 15.6 years old, 13.6 years old, and 15.7 years old. The duration between the onset of the first symptoms and diagnosis was 3.9 years, 6.1 years, and 11.2 years, respectively.

### Adjunctive treatments, ERT, and follow-up

Comprehensive monitoring at regular intervals and health education (such as avoidance of physical exertion or exposure to circumstances that provoke attacks) were carried out for these children and their families. Adjunctive treatments, including angiotensin enzyme converting (ACE) inhibitors or angiotensin receptor blockers (ARBs), neuroleptic drugs, and psychiatric referrals, were provided accordingly (Table 1).

Four of the diagnosed FD children started to receive ERT (agalsidase beta 1 mg/kg, every 2 weeks): (1) Patient ID 08 received ERT beginning in August 2020 along with ARB, carbamazepine, ketosteril, calcitriol, and febuxostat. After 11 months of ERT combined with adjunctive treatment, Lyso-GL-3 dropped from 76 ng/ml to 19 ng/ml, the stage of chronic kidney disease was stable at stage 3, and 24-h urine protein decreased from 4.47 g to 2.46 g. (2) Patient ID 04 received ERT from January 2021 along with oxcarbazepine. After 6 months of treatment, Lyso-GL-3 dropped from 93 ng/ml to 11 ng/ml. (3) Patient ID 09 and Patient ID 10 started ERT one week and two weeks ago, respectively.

## Discussion

In this study, based on a defined screening protocol (DBS triple-test for children with a high-risk profile of FD and newborn screening by genetic testing) and a multidisciplinary approach, we newly diagnosed 7 children, including 2 neonates, with FD in the hospital in the last twelve months accompanied by 3 referred FD children from other hospitals. No case was diagnosed with FD before the establishment of the MDT approach. Five children were diagnosed with FD in a timely manner by high-risk profile and DBS triple-test screening, with a yield of diagnosis of 14.3%. After MDT evaluation, four of them started to receive ERT, and the accumulation of lyso-GL-3 in the blood was significantly improved, which could delay vital organ pathology and disease progression.

From a historical point of view, the first teams that began patient care using a multidisciplinary approach were oncologists. It is now a widely held view that the treatment of most cancers has benefitted from this integrated MDT approach, and patient satisfaction and efficiency are improved [13]. FD is considered a rare disease. The diagnosis is challenging due to variability and complexity in the presentation and the chronic, slowly progressive nature of FD. It is recognized that there are many individuals affected who are unscreened and undiagnosed. A multidisciplinary approach is advocated to screen, diagnose, care for, and manage patients with FDs, and the United States-based perspective also recommends multidisciplinary clinical teams to oversee the management of FD in children [5]. Since FD remains poorly recognized in children in China and no case had been diagnosed in our hospital before, it was urgent to set up the MDT for pediatric FD in the hospital, which included the screening team who focused on high-risk profiles and the management team who focused on the diagnosis, treatment, and follow-up. To make more staff aware of this disease, continuous education was given not only to MDT members but also to others from associated departments.

A longitudinal study from Italy that screened ~ 17,000 individuals with clinical manifestations suggestive of FD showed that 100% of males with classic FD had α-Gal A enzymatic activity below the normal reference range, in contrast to only 46% of females harbouring the same pathogenic variants [4]. A similar result was demonstrated in a United States study that showed that α-Gal A activity in DBS had high sensitivity but lower specificity for FD in males, as not all males with low α-Gal A activities were confirmed to have FD [12]. Therefore, using *GLA* sequencing and Lyso-GL-3 detection was useful for disease confirmation in males. For females, they found that first-tier testing consisting of *GLA* sequencing and Lyso-GL-3 analysis provided the greatest sensitivity and specificity, whereas enzyme testing had lower sensitivity and was therefore less useful as a first-tier test [9]. Accordingly, we set up the DBS triple-test (α-Gal A, Lyso-GL-3, *GLA* gene) screening approach using sex-specific algorithms. For boys, a level of α-Gal A determined by MS/MS below normal was followed by Sanger sequencing of the *GLA* gene and the level of lyso-GL-3 tested by MS/MS. For girls, a level of lyso-GL-3 tested by MS/MS above normal was followed by Sanger sequencing of the *GLA* gene, and the level of α-Gal A was determined by MS/MS. Sex-specific detection algorithms might prioritize tests with high specificity and sensitivity that offer an effective way to identify individuals with FD.

Of course, children with FD may initially experience pain, hypohidrosis, and gastrointestinal symptoms, while renal and cardiac disease may present later in adolescence or adulthood [2]. A hallmark sign of classic FD in children is neuropathic pain in the hands and feet, most commonly in the palms, soles and fingertips. This symptom was reported by up to 72.3% of patients with FD and is more frequently present in boys [5]. The mean age of onset of pain was reported as 10 years in boys and 15 years in heterozygous girls [5]. In this study, thirty-five high-risk children were referred for screening, with a yield of diagnosis of 14.3% (5/35). Among these 5 diagnosed children, four had neuropathic pain. One of them had pain accompanied by abnormal liver function. Although abnormal liver function was unexpected in FD, previous study showed that liver biopsy from a longstanding FD patient with abnormal liver function indicated the accumulation of GL-3 in liver [14]. Whether the abnormal liver function is related to the deposition of GL-3 and lyso-GL-3, or is caused by other reasons, further follow-up and more cases are needed to clarify this issue. In addition, the other diagnosed child with FD in this study was screened due to a high-risk profile of unexplained renal tubular dysfunction. In FD patients, renal involvement is caused by the accumulation of GL-3 and Lyso-GL-3 in all renal cell types, including podocytes, endothelial cells, mesangial cells and tubular cells [15, 16]. Although less common as tubular dysfunction, manifestations include Fanconi syndrome, distal renal tubular acidosis, and isosthenuria [15, 17]. Tubular damage and dysfunction may be accompanied by the excretion of tubular lesion markers, such as α1-microglobulin and retinol-binding protein [15].

In FD, based on enzymatic assays for α-Gal A activity, previous newborn screening studies revealed frequencies of the classic and later-onset phenotypes of up to 1 in 22,570 males and 1 in 1,390 males, respectively [3]. It allows for the identification and monitoring of individuals with FD from an early age and identifies affected adults in the family. Such programs have been initiated in some states in the United States [18] and several European countries [19, 20]. In this study, we enrolled newborns who were at risk of genetic metabolic disorders undergoing genetic testing in the hospital, and the *GLA* gene was listed as a routine analysis gene. Two neonates were detected early with *GLA* mutations in the 12-month period, with a yield of detection of 0.14% (2/1420). This newborn screening approach needs further evaluation and cost-effectiveness analysis to identify its feasibility and value. Newborn screening raises challenges in defining the most appropriate way to counsel families of infants diagnosed with FD and how to effectively monitor and manage those infants to optimize clinical outcomes [21]. Although the use of newborn screening for the identification of FD remains an ethical issue, previous studies indicate that most patients prefer to be informed [21]. Furthermore, families with known FD can be offered preimplantation genetic diagnosis (PGD) of embryos prior to implantation during assisted reproduction.

In addition, ERT, adjunctive treatments, and regular follow-up can provide significant clinical benefit. There is strong circumstantial evidence and increasing clinical recognition of the crucial importance of early treatment initiation to mitigate the long-term impact of the disease [22]. The management of FD requires a coordinated, multidisciplinary care approach. Asymptomatic children should be followed closely for the development of renal, cardiac, neurological, or gastrointestinal signs, symptoms, or laboratory changes, which would warrant treatment initiation [6]. A previous study indicated that after 65 months of treatment with a 1 mg/kg agalsidase dose every other week, patients had substantial clearance of podocyte GL-3 inclusions [23]. The greatest clearance was observed in the youngest patient treated, beginning at age 7 years. ERT treatment during childhood may positively impact school attendance, exercise performance, energy levels, and pain, with subsequent improvements in quality of life, and early treatment before the onset of potentially irreversible vital organ pathology is ideal.

Some limitations of our study need to be acknowledged. First, this multidisciplinary approach to the screening and management of children with FD was only implemented in the last twelve months. Second, in this study, we used the DBS triple-test screening approach with sex-specific algorithms, but the sensitivity and specificity of this method were not verified in our population. Third, although missense and nonsense variants account for the majority of disease-causing mutations in FD, Sanger sequencing of the *GLA* gene could not detect insertions, deletions, and structural rearrangements.

In conclusion, early diagnosis of patients with FD is vital. A year after establishing an MDT for pediatric FD, this program met its goals. Screening and management of children with FD was effective based on a defined screening protocol and a multidisciplinary approach. We should pay more attention to the high-risk profiles of pain, angiokeratoma, decreased sweating, and unexplained chronic kidney disease in children. In this sense, this model could be extended to other regions in China.

## Data Availability

All data generated or analysed during this study are included in this published article.

## Abbreviations

α-Gal A: α-Galactosidase A
ACEI: Angiotensin-converting enzyme inhibitor
ACMG: American College of Medical Genetics and Genomics
Aff.: Affected
ARB: Angiotensin receptor blocker
DBS: Dried blood spots
ERT: Enzyme replacement therapy
F: Father
FD: Fabry disease
Hemi: Hemizygous
Het: Heterozygous
ID: Individual identifier
Lyso-GL-3: Globotriaosylsphingosine (deacylated form of GL-3)
LP: Likely pathogenic
LSD: Lysosomal storage disorder
M: Mother
MDT: Multidisciplinary team
NA: Not available
P: Pathogenic
PGD: Preimplantation genetic diagnosis
TES: Targeted exome sequencing
VUS: Variant of uncertain clinical significance
WES: Whole exome sequencing
WT: Wild type
